# Enlargement of the knowledge of *Cortinarius* section *Anomali* (Agaricales, Basidiomycota): introducing three new species from China

**DOI:** 10.3389/fcimb.2023.1215579

**Published:** 2023-06-12

**Authors:** Qiu-Yue Zhang, Can Jin, Hong-Min Zhou, Zi-Yan Ma, Yi-Zhe Zhang, Jia-Qi Liang, Jing Si, Hai-Jiao Li

**Affiliations:** ^1^ Institute of Microbiology, School of Ecology and Nature Conservation, Beijing Forestry University, Beijing, China; ^2^ National Institute of Occupational Health and Poison Control, Chinese Center for Disease Control and Prevention, Beijing, China

**Keywords:** fungi diversity, morphology, new taxa, phylogeny, taxonomy

## Abstract

*Cortinarius* is a globally distributed agaricoid genus that has been well studied in Europe and America with over 1,000 described species. However, as part of an ongoing effort to investigate the diversity of *Cortinarius* section *Anomali* in China, the resource investigation and classification research are still limited, and the species diversity has not been clarified by far. During the re-examination of the Chinese *Cortinarius* specimens, *C. cinnamomeolilacinus*, *C. subclackamasensis*, and *C. tropicus*, belonging to the sect. *Anomali*, were described in China as new to science based on morphological examination and phylogenetic analysis. The three new species are described and illustrated in detail according to the Chinese materials. The phylogenetic analysis based on internal transcribed spacer sequences confirmed the placement of the three species in the *Cortinarius* sect. *Anomali* clade. Phylogenetically related and morphologically similar species to these three new species are discussed.

## 1 Introduction

*Cortinarius* (Pers.) Gray, established based on *Cortinarius violaceus* (L.) Gray, is the largest genus of Agaricales with a worldwide distribution (Clements and Shear, 1931; Garnica et al., 2016; Varga et al., 2019). It is mainly marked by a fugacious veil enveloping the basidiocarp and a cortina, which initially covers the lamellae, but later vanishes in expanding basidiocarps (Stensrud et al., 2014). Macroscopically, members of this genus are highly variable, with their basidiocarps, lamellae, and basidiospores varying considerably in size, shape, or color (Peintner et al., 2004; Frøslev et al., 2006; Niskanen et al., 2009; Niskanen et al., 2013; Liimatainen et al., 2014; Liimatainen et al., 2015; Niskanen et al., 2016). *Cortinarius* species are widely reported in temperate and subtropical forests and form mycorrhizal associations with ectotrophic trees, such as plants of the Cistaceae, Fagaceae, Malvaceae, Nothofagaceae, Pinaceae, and Salicaceae families (Singer, 1986; Frøslev et al., 2006; Soop et al., 2018). With the advances in taxonomy and molecular biology techniques, increases have been detected in the number of species in the genus *Cortinarius*. To date, more than 5,000 scientific names in the genus have been published as listed in the Index Fungorum (http://www.indexfungorum.org/Names/names.asp, 2023), and about 2,000 species are estimated in the Dictionary of Fungi, 10th edition (Ammirati et al., 2007; Kirk et al., 2008; Brandrud et al., 2014).

Owing to the considerable morphological variations in this genus, the subdivision of *Cortinarius* into subgeneric units has caused some problems (Peintner et al., 2004). Morphologically, *Cortinarius* has been divided into several different subgenera and infrageneric sections by various taxonomists, which results in taxonomic chaos and indicates that morphology alone is insufficient for recognizing natural units in this group of fungi (Garnica et al., 2005; Garnica et al., 2009). In recent research, phylogenetic analyses of the genus have contributed to the delimitation of taxonomic entities within the genus (Peintner et al., 2003; Suarez-Santiago et al., 2009; Dima et al., 2016; Dima et al., 2021).

*Cortinarius* sect. *Anomali* Konrad & Maubl., a species-rich group, is established based on *Cortinarius anomalus* (Fr.) Fr. It is characterized by a telamonioid/sericeocyboid appearance, often with yellowish to brownish universal veil remnants on the stipe, typically young bluish lamellae, and subglobose to broadly ellipsoid or rarely ellipsoid, verrucose spores (Dima et al., 2016; Dima et al., 2021). *Anomali* was originally placed by Brandrud et al. (1989) as a section of subgenus *Telamonia* Melot but not belong to the subgenus *Telamonia* s. str. based on later phylogenetic data (Høiland and Holst-Jensen, 2000; Peintner et al., 2004; Garnica et al., 2005; Niskanen et al., 2009). Later, many species were added or transferred to sect. *Anomali*, causing confusions in the classification of sect. *Anomali*. No consensus on the content or placement of this section have been reached to date (Bidaud et al., 1992; Bidaud et al., 1994; Consiglio et al., 2005; Consiglio et al., 2006; Consiglio, 2012; Dima et al., 2016). For instances, *C. spilomeus* (Fr.) Fr. and *C. bolaris* (Pers.) Fr. have been included in the section, and sometimes have been separated in another sect. *Spilomei* (Moënne-Locc. & Reumaux) Consiglio, D. Antonini & M. Antonini (Dima et al., 2016). Some phylogenetic studies show that sect. *Anomali* is a monophyletic group in the genus *Cortinarius*, without any traditional subgenera (Dima et al., 2016; Dima et al., 2021). The classification of sect. *Anomali* has been studied previously and systematically in Europe and North America, but rarely in Asia and Africa (Kauffman, 1905; Murrill, 1946; Dima et al., 2016; Ammirati et al., 2017). In China, about 163 *Cortinarius* species, including dozens of new species, have been described in the past 10 years, with only three species belonging to the sect. *Anomali* (Wei and Yao, 2013; Xie et al., 2019; Luo and Bau, 2021; Xie et al., 2021; Xie, 2022). On this basis, more taxa of the genus are waiting to be discovered in China.

In this study, we conducted taxonomic and phylogenetic studies of *Cortinarius* sect. *Anomali* in China. Three new species were found during the intensive fieldwork and are described here based on their morphological and ecological characteristics, as well as phylogenetic evidence.

## 2 Materials and methods

### 2.1 Morphological studies

All specimens have been deposited in the National Institute of Occupational Health and Poison Control, Chinese Center for Disease Control (NIOHP, China CDC). Macro-morphological descriptions were based on field notes and dried specimens. Microscopic features were examined and described in 5% KOH, Congo Red, or Melzer’s reagent and observed under a Nikon Eclipse 80i microscope (Nikon, Tokyo, Japan) with a magnification of up to ×1,000. Thirty basidiospores were measured per collection (excluding apiculus and ornamentation), and the averages (av. X) and quotients (av. *Q* = L/B) were calculated. Color terms are cited from Anonymous (1969) as well as Kornerup and Wanscher (1978).

### 2.2 DNA extraction and sequencing

A Phire^®^ Plant Direct PCR Kit (Finnzymes Oy, Finland) was used to obtain PCR products from dried specimens, according to the manufacturer’s instructions and as described previously by Li et al. (2015), with some modifications. The following primer pairs were used to amplify the internal transcribed spacer (ITS): ITS5 (5′‐GGA AGT AAA AGT CGT AAC AAG G‐3′) and ITS4 (5′‐TCC TCC GCT TAT TGATAT GC‐3′) (White et al., 1990). The PCR procedure was as follows: initial denaturation at 98 °C for 5 min, followed by 35 cycles at 98 °C for 5 s, 58 °C for 5 s, and 72 °C for 5 s, and a final extension of 72 °C for 10 min. The PCR products were purified and sequenced by Sangon Biotech, China. The newly generated sequences from this study have been deposited in GenBank and are listed in **Table 1**.

**Table 1:** Taxa information and GenBank accession numbers of the sequences used in this study.

Species	Specimen no.	Locality	Section	ITS no.
*Cortinarius albidipes*	WTU: JFA12420	Colorado, US	*Anomali*	MZ580486
*C. albidipes*	NYS-F-000129 (holotype)	New York, US	*Anomali*	MZ580485
*C. albidipes*	MQ18-CMMF001826	Québec, Canada	*Anomali*	MN750945
*C. albidipes*	HRL0614	Québec, Canada	*Anomali*	KJ705108
*C. albidipes*	CNV98	New Hampshire, US	*Anomali*	MT345274
*C. albidoavellaneus*	MICH10313 (holotype)	Michigan, US	*Anomali*	MZ580483
*C. albocyaneus*	CFP1482	Italy	*Anomali*	KX302202
*C. albocyaneus*	CFP1177 (epitype)	Sweden	*Anomali*	KX302206
*C. albocyaneus*	NYS-F-000864 (holotype)	New York, US	*Anomali*	MZ580482
*C. albomalus*	iNAT59505932	New Jersey, US	*Anomali*	MW305253
*C. albomalus*	H7000816 (holotype)	Ontario, Canada	*Anomali*	MZ568645
*C. albomalus*	HRL2777	Ontario, Canada	*Anomali*	MN751632
*C. anocorium*	H7068022 (holotype)	Florida, US	*Anomali*	MZ568646
*C. anomalodelicatus*	TN11-241	Alaska, US	*Anomali*	MZ580481
*C. anomalodelicatus*	JFA8146 (holotype)	Colorado, US	*Anomali*	MZ580480
*C. anomalomontanus*	JFA9919 (holotype)	Wyoming, US	*Anomali*	MZ580478
*C. anomalomontanus*	JFA9973	Wyoming, US	*Anomali*	MZ580479
*C. anomalopacificus*	JFA11887	California, US	*Anomali*	MZ580471
*C. anomalopacificus*	DBB11745 (holotype)	California, US	*Anomali*	MZ663774
*C. anomalopacificus*	DBB27748	California, US	*Anomali*	MZ663775
*C. anomalopacificus*	TN12-271	California, US	*Anomali*	MZ663776
*C. anomalopacificus*	TN12-301	California, US	*Anomali*	MZ663777
*C. anomalopacificus*	TN12-093	California, US	*Anomali*	MZ580473
*C. anomalopacificus*	TN12-074	California, US	*Anomali*	MZ580469
*C. anomalopacificus*	TN12-253	California, US	*Anomali*	MZ580468
*C. anomalopacificus*	TN12-091	California, US	*Anomali*	MZ580472
*C. anomalopacificus*	TN12-161	California, US	*Anomali*	MZ580474
*C. anomalopacificus*	TN12-164	California, US	*Anomali*	MZ580475
*C. anomalovelatus*	JFA13109 (holotype)	Washington, US	*Anomali*	FJ717605
*C. anomalovelatus*	DBB23800	Oregon, US	*Anomali*	MZ663776
*C. anomalovelatus*	PK4741	British Columbia, Canada	*Anomali*	FJ039655
*C. anomalovelatus*	JFA13109 (holotype)	Washington, US	*Anomali*	KJ019014
*C. anomalus*	NL-5414	Massachusetts, US	*Anomali*	MZ663777
*C. anomalus*	CNV9	New Hampshire, US	*Anomali*	MT345186
*C. anomalus*	MQ18-HL1492-QFB30079	Québec, Canada	*Anomali*	MN750971
*C. anomalus*	TENN067720	North Carolina, US	*Anomali*	MZ663778
*C. anomalus*	TENN067730	North Carolina, US	*Anomali*	MZ663779
*C. anomalus*	CFP1154 (neotype)	Sweden	*Anomali*	KX302224
*C. barlowensis*	TN07-366	Washington, US	*Anomali*	KJ019015
*C. barlowensis*	MN	British Columbia, Canada	*Anomali*	FJ157009
*C. barlowensis*	JFA13140 (holotype)	Washington, US	*Anomali*	FJ717554
*C. bolaris*	3861	Québec, Canada	*Bolares*	KJ705110
*C. bolaris*	CFP1008 (neotype)	Sweden	*Bolares*	KX302233
*C. bolaris*	TENN61650	Tennessee, US	*Bolares*	FJ596851
*C. brevissimus*	Cort H2QY2	New York, US	*Anomali*	JX030219
*C. brevissimus*	NYS-F-000541 (holotype)	New York, US	*Anomali*	MZ580467
*C. caeruleoanomalus*	JFA13084 (holotype)	Tennessee, US	*Anomali*	MZ663780
*C. caeruleoanomalus*	TENN068383	North Carolina, US	*Anomali*	KY744156
*C. caesiellus*	MICH10325 (holotype)	Michigan, US	*Anomali*	MZ580484
*C. caesiifolius*	SAT13-298-15	Oregon, US	*Anomali*	MZ048733
*C. caesiifolius*	MICH10326 (holotype)	Washington, US	*Anomali*	MZ580462
*C. caesiifolius*	TN12-136	California, US	*Anomali*	MZ580465
*C. caesiifolius*	TN07-489	Washington, US	*Anomali*	MZ580466
*C. caesiifolius*	DBB37600	Minnesota, US	*Anomali*	MZ663781
*C. caesiifolius*	JMB10-20-2007-15	Washington, US	*Anomali*	FJ717517
*C. caesiifolius*	TN12-066	California, US	*Anomali*	MZ580463
*C. caesiifolius*	TN12-136	California, US	*Anomali*	MZ580465
*C. caesiifolius*	TN12-118	California, US	*Anomali*	MZ580464
*C. camphoratus*	EH23	British Columbia, Canada	*Camphorati*	FJ717505
*C. caninus*	JFA7985	Ontario, Canada	*Anomali*	MZ580454
*C. caninus*	NS18	California, US	*Anomali*	MZ663782
*C. caninus*	JFA10347	Wyoming, US	*Anomali*	MZ580459
*C. caninus*	JFA9425	Wyoming, US	*Anomali*	MZ580461
*C. caninus*	JFA12434	Wyoming, US	*Anomali*	MZ580456
*C. caninus*	JFA9470	Wyoming, US	*Anomali*	MZ580457
*C. caninus*	JFA10348	Wyoming, US	*Anomali*	MZ580460
*C. caninus*	JFA8009	Minnesota, US	*Anomali*	MZ580455
*C. caninus*	JFA9920	Wyoming, US	*Anomali*	MZ580458
*C. caninus*	CFP627 (epitype)	Sweden	*Anomali*	KX302250
*C. caninus*	TH4Cc	British Columbia, Canada	*Anomali*	KF753582
***C. cinnamomeolilacinus***	**Li 140805-18**	**Yunnan, China**	***Anomali***	OQ913389*
***C. cinnamomeolilacinus***	**tcmb 005**	**Yunnan, China**	***Anomali***	OQ913388*
***C. cinnamomeolilacinus***	**TCWH 007 (holotype)**	**Yunnan, China**	***Anomali***	OQ913384*
***C. cinnamomeolilacinus***	**LLLJ 20170805-002**	**Yunnan, China**	***Anomali***	OQ913386*
***C. cinnamomeolilacinus***	**WX 20170922065**	**Yunnan, China**	***Anomali***	OQ913392*
***C. cinnamomeolilacinus***	**LYWF015**	**Yunnan, China**	***Anomali***	OQ913387*
***C. cinnamomeolilacinus***	**Li 180825-21**	**Yunnan, China**	***Anomali***	OQ913390*
***C. cinnamomeolilacinus***	**YNLF20220814-155**	**Yunnan, China**	***Anomali***	OQ913393*
***C. cinnamomeolilacinus***	**Li 130908-29**	**Yunnan, China**	***Anomali***	OQ913391*
***C. cinnamomeolilacinus***	**Li 161015-10**	**Guizhou, China**	***Anomali***	OQ913385*
*C. clackamasensis*	JFA11616 (holotype)	Oregon, US	*Anomali*	MZ580452
*C. clackamasensis*	OSC114858	Oregon, US	*Anomali*	EU669315
*C. clackamasensis*	TN11-451	Washington, US	*Anomali*	MZ580453
*C. clackamasensis*	OSC109672	Oregon, US	*Anomali*	EU652360
*C. clintonianus*	DBB21645	British Columbia, Canada	*Anomali*	MZ663783
*C. clintonianus*	JFA8329	Ontario, Canada	*Anomali*	MZ580451
*C. clintonianus*	MIN896348	Minnesota, US	*Anomali*	MZ663784
*C. clintonianus*	NYS-F-000786 (holotype)	New York, US	*Anomali*	MZ580450
*C. clintonianus*	MQ18-CMMF002618	Québec, Canada	*Anomali*	MN751121
*C. clintonianus*	136C	Michigan, US	*Anomali*	FJ769528
*C. clintonianus*	SDL13	British Columbia, Canada	*Anomali*	KM403009
*C. deceptivus*	iNAT56430786	New York, US	*Anomali*	MT939445
*C. deceptivus*	NL-5180	New York, US	*Anomali*	MZ663785
*C. deceptivus*	WTU-F-69333	New Hampshire, US	*Anomali*	MZ663786
*C. deceptivus*	WTU-F-69313	Massachusetts, US	*Anomali*	MZ663787
*C. deceptivus*	MICH10343 (syntype)	New York, US	*Anomali*	MZ663788
*C. durifoliorum*	PDD101829 (holotype)	New Zealand	*Anomali*	KJ635210
*C. dysodes*	PDD70499 (holotype)	New Zealand	*Camphorati*	GU233340
*C. epsomiensis*	K(M)74963 (holotype)	UK	*Anomali*	MK010952
*C. epsomiensis*	TN06-165	Finland	*Anomali*	KX302258
*C. eunomalus*	PDD94040 (holotype)	New Zealand	incertae sedis	JQ287690
*C. ferrusinus*	JB8106 13 (holotype)	Spain	*Spilomei*	KY657254
*C. harvardensis*	NL-5415 (holotype)	Massachusetts, US	*Anomali*	MZ663789
*C. harvardensis*	MQ18-HL1449-QFB30070	Québec, Canada	*Anomali*	MN751560
*C. harvardensis*	MQ17058-QFB29566	Québec, Canada	*Anomali*	MN751559
*C. harvardensis*	clone ads9.e	Nova Scotia, Canada	*Anomali*	MK131480
*C. holophaeus*	UBC-F17161	British Columbia, Canada	*Anomali*	GQ159904
*C. holophaeus*	UBC-F17157	British Columbia, Canada	*Anomali*	GQ159900
*C. huddartensis*	DBB12118 (holotype)	California, US	*Anomali*	MZ663790
*C. huddartensis*	src174	California, US	*Anomali*	DQ974719
*C. ionomataius*	PDD89089 (holotype)	New Zealand	incertae sedis	GU222303
*C. jonimitchelliae*	HL03-339 (holotype)	Sweden	*Anomali*	KX302253
*C. kranabetteri*	TN11-287 (holotype)	Alberta, Canada	*Anomali*	MZ580449
*C. kranabetteri*	UBC-F16436	British Columbia, Canada	*Anomali*	FJ039657
*C. kranabetteri*	UBC-F16435	British Columbia, Canada	*Anomali*	FJ039656
*C. latiodistributus*	JFA13487	Washington, US	*Anomali*	MZ663793
*C. latiodistributus*	YM187	Japan	*Anomali*	AB848436
*C. latiodistributus*	OSC115143	Washington, US	*Anomali*	EU652359
*C. latiodistributus*	DB6359	Norway	*Anomali*	MZ663792
*C. latiodistributus*	TN02-490	Finland	*Anomali*	MZ580448
*C. latiodistributus*	SMIA46	British Columbia, Canada	*Anomali*	FJ039658
*C. latiodistributus*	OSC114595	Washington, US	*Anomali*	EU837213
*C. latiodistributus*	DB6139 (holotype)	Sweden	*Anomali*	MZ663791_
*C. latiodistributus*	SMI16	British Columbia, Canada	*Anomali*	FJ157134
*C. lepidopus*	DB6253	Hungary	*Anomali*	MZ663794
*C. lepidopus*	170817-24	Heilongjiang, China	*Anomali*	OQ913382*
*C. lepidopus*	170817-29	Heilongjiang, China	*Anomali*	OQ913383*
*C. lividomalvaceus*	JMT-15102001 (holotype)	France	*Anomali*	KY315416
*C. modestus*	TN10-035	Québec, Canada	*Anomali*	MZ580447
*C. modestus*	MQ17140-QFB29648	Québec, Canada	*Anomali*	MN751561
*C. modestus*	NYS-F-001966 (holotype)	New York, US	*Anomali*	MZ580446
*C. modestus*	MQ17272-QFB29780	Québec, Canada	*Anomali*	MN751565
*C. modestus*	MQ18-HL0629-QFB30005	Québec, Canada	*Anomali*	MN751564
*C. modestus*	2313-QFB25737	Québec, Canada	*Anomali*	KJ705109
*C. nettieae*	JFA9613 (holotype)	Washington, US	*Anomali*	MZ580442
*C. nettieae*	JFA8747	Oregon, US	*Anomali*	MZ580443
*C. nettieae*	TN09-167	Oregon, US	*Anomali*	MZ580444
*C. nettieae*	TN09-176	Oregon, US	*Anomali*	MZ580445
*C. nettieae*	DAVFP27503	British Columbia, Canada	*Anomali*	EU821675
*C. ochraceodiscus*	DJM2195 (holotype)	Minnesota, US	*Anomali*	MZ663795
*C. ochraceodiscus*	DJM2194	Minnesota, US	*Anomali*	MZ663796
*C. pelerinii*	XC2012-21 (holotype)	France	*Anomali*	MH784627
*C. perrotensis*	TENN071126 (holotype)	Québec, Canada	*Anomali*	KX897405
*C. perviolaceus*	FN05_2	New York, US	*Anomali*	KU878589
*C. perviolaceus*	HBK-M11-2	Tennessee, US	*Anomali*	MG982536
*C. perviolaceus*	FLAS-F61753	Florida, US	*Anomali*	MH281882
*C. perviolaceus*	JFA9132	Florida, US	*Anomali*	MZ580441
*C. perviolaceus*	JFA9124	Florida, US	*Anomali*	MZ580439
*C. perviolaceus*	FLAS-F32992 (holotype)	Florida, US	*Anomali*	MZ580438
*C. perviolaceus*	JFA13070	Tennessee, US	*Anomali*	MZ663799
*C. perviolaceus*	JFA9128	Florida, US	*Anomali*	MZ580440
*C. perviolaceus*	3Bart56R	New Hampshire, US	*Anomali*	HQ022110
*C. perviolaceus*	NL-5173	Massachusetts, US	*Anomali*	MZ663798
*C. perviolaceus*	FLAS-F61648	Florida, US	*Anomali*	MH212024
*C. perviolaceus*	WU-Myc 44566	Georgia, US	*Anomali*	MZ663797
*C. perviolaceus*	FLAS-MES-2177	Florida, US	*Anomali*	MT415970
*C. putorius*	TN12-230	California, US	*Camphorati*	KR011123
*C. rarus*	JLF8707	Oregon, US	*Anomali*	MW341328
*C. rarus*	JLF8771	Oregon, US	*Anomali*	MW341331
*C. rarus*	JLF3304	California, US	*Anomali*	MF135162
*C. rarus*	ADP-140531–1	Washington, US	*Anomali*	MZ663801
*C. rarus*	DBB04712 (holotype)	California, US	*Anomali*	MZ663800
*C. rattinoides*	PDD88283 (holotype)	New Zealand	*Anomali*	JX000375
*C. sclerophyllarum*	HO-A20430A6 (paratype)	Australia	*Anomali*	AY669637
*C. sericeolazulinus*	JFA12053 (holotype)	Costa Rica	*Anomali*	EF420146
*C. spilomeus*	CFP1137 (neotype)	Sweden	*Spilomei*	KX302267
*C. spilomeus*	SMI297	British Columbia, Canada	*Spilomei*	FJ039659
***C. subclackamasensis***	**BJMTG20170830-34**	**Beijing, China**	***Anomali***	OQ913395*
***C. subclackamasensis***	**20190822-11**	**Hebei, China**	***Anomali***	OQ913396*
***C. subclackamasensis***	**Li 170818-16 (holotype)**	**Heilongjiang, China**	***Anomali***	OQ913394*
***C. subclackamasensis***	**Li 170818-01**	**Heilongjiang, China**	***Anomali***	OQ913397*
***C. subclackamasensis***	**YJ4**	**China**	***Anomali***	**OM867684**
***C. subclackamasensis***	**HBAU15437**	**China**	***Anomali***	**MW862347**
***C. subclackamasensis***	**HBAU15679**	**China**	***Anomali***	**MZ145077**
***C. subclackamasensis***	**HBAU15672**	**China**	***Anomali***	**MZ145076**
***C. subclackamasensis***	**110116MFBPC490**	**China**	***Anomali***	**MW554249**
*C. suecicolor*	PDD74698 (holotype)	New Zealand	*Anomali*	JX000360
*C. tabularis*	CFP949 (epitype)	Sweden	*Anomali*	KX302275
*C. tabularis*	IK98-1190	Finland	*Anomali*	KX302269
*C. tabularis*	TN11-219	Alaska, US	*Anomali*	MZ580437
*C. tasmacamphoratus*	HO A20606A0	Tasmania, Australia	*Camphorati*	AY669633
*C. tasmacamphoratus*	BH2055F	Tasmania, Australia	*Camphorati*	JF960672
*C. tetonensis*	ME12-B10	Alaska, US	*Anomali*	JX436875
*C. tetonensis*	ME12-B4	Alaska, US	*Anomali*	JX436874
*C. tetonensis*	ME12-D3	Alaska, US	*Anomali*	JX436876
*C. tetonensis*	JFA10350 (holotype)	Wyoming, US	*Anomali*	MZ580436
*C. tetonensis*	JFA10349	Wyoming, US	*Anomali*	U56024
*C. tetonensis*	36_N343	Svalbard, Norway	*Anomali*	HQ445618
*C. tristis* s. Garnica	TUB011917	Chile	*Anomali*	AY669648
***C. tropicus***	**Li 150728-63**	**Yunnan, China**	***Anomali***	OQ913381*
***C. tropicus***	**tcqushi006 (holotype)**	**Yunnan, China**	***Anomali***	OQ913379*
***C. tropicus***	**Li 150728-56**	**Yunnan, China**	***Anomali***	OQ913380*
*Cortinarius* aff. *nettiae*	MQ17280-QFB29788	Québec, Canada	*Anomali*	MN750925
*Cortinarius* aff. *nettiae*	MQ17300-QFB29808	Québec, Canada	*Anomali*	MN750926
*Cortinarius* LHJ sp. 1	SC20170921-025	Guizhou, China	*Anomali*	OQ920005*
*Cortinarius* LHJ sp. 2	190527-01	Tibet, China	*Anomali*	OQ920004*
*Cortinarius* LHJ sp. 3	170805-35	Yunnan, China	*Anomali*	OQ920003*
*Cortinarius* sp.	Pj3-mOTU024	Japan	*Anomali*	LC260432
*Cortinarius* sp. 1	7-70M6	California, US	*Anomali*	JQ393041
*Cortinarius* sp. 2	YM73	Japan	*Anomali*	LC175532
*Cortinarius* sp. 3	GO-2010-171	Mexico	*Anomali*	KC152091
*Cortinarius* sp. 4	F18506	British Columbia, Canada	*Anomali*	FJ157104
*Cortinarius* sp. 5	TN10-141	Québec, Canada	*Anomali*	MZ821030
*Cortinarius* sp. 6	OUC97234	British Columbia, Canada	*Anomali*	DQ097877
*Cortinarius* sp. 6	HRL1598-QFB32934	Québec, Canada	*Anomali*	MW845268
*Cortinarius* sp. 7	YM1162	Japan	*Anomali*	LC175062
*Cortinarius* sp. 8	OUC97199	British Columbia, Canada	*Anomali*	DQ093855
*Cortinarius* sp. 9	JLP2431	Oregon, US	*Anomali*	DQ377379
*Cortinarius* sp. 10	QFB28611	Québec, Canada	*Anomali*	MN992356
*Cortinarius* sp. 11	Pdmt24	Japan	*Anomali*	AB251830
*Cortinarius* sp. 12	HV_D8	Alaska, US	*Anomali*	JX630733
*Cortinarius* sp. 12	ME12-D2	Alaska, US	*Anomali*	JX436862
*Cortinarius* sp. 12	MEN-JG-096	Svalbard, Norway	*Anomali*	JF304376
*Cortinarius* sp. 13	Russell iNaturalist 8602253	Indiana, US	*Anomali*	MZ710565
*Cortinarius* sp. 13	YM873	Japan	*Anomali*	AB848465
*Cortinarius* sp. 15	MQ21-HRL2477-QFB32937	Québec, Canada	*Anomali*	MW845269
*Cortinarius* sp. 15	PERTH06437109	Australia	*Anomali*	MG553013
*Cortinarius* sp. 16	PDD10596	New Zealand	*Anomali*	MH101576
*Cortinarius* sp. 17	NVE433	Colombia	*Anomali*	KF937326
*Cortinarius* sp. 17	NVE219	Colombia	*Anomali*	KF937328
*Cortinarius* sp. 18	PERTH06659462	Australia	*Anomali*	MG553083
*Cortinarius* sp. 19	BH3573F	Tasmania, Australia	*Camphorati*	JF960738
*Cortinarius* sp. 20	SWUBC741	British Columbia, Canada	*Spilomei*	DQ481671
*Cortinarius* sp. 20	WUBC747	British Columbia, Canada	*Spilomei*	DQ481752
*Cortinarius* sp. 21	PDD:107722	New Zealand	incertae sedis	KT875175

New species are in bold. Newly generated sequences are marked with asterisk (*).

### 2.3 Phylogenetic analysis

New sequences generated in this study and additional sequences retrieved from GenBank (**Table 1**) were aligned using BioEdit 7.0.5.3 (Hall, 1999) and ClustalX 1.83 (Thompson et al., 1997), followed by manual adjustments. Sequences of *Cortinarius bolaris* (sect. *Bolares*) were used as outgroups (Hughes et al., 2009; Dima et al., 2021). The maximum likelihood (ML) and Bayesian inference (BI) methods were used for the phylogenetic analysis. The best-fit model was selected by ModelFinder (Kalyaanamoorthy et al., 2017), adopting Akaike information criterion (AIC). The ML analysis was carried out using RAxML 8.2.12 (Stamatakis, 2006; Silvestro and Michalak, 2012), and the BI tree reconstruction was reconstructed using MrBayes 3.2.5 (Ronquist et al., 2012). Four Markov chains were run for two runs from random starting trees for 10 million generations, and the trees were sampled every 1,000 generations. The burn-in was set to discard 25% of the trees. A majority rule consensus tree of all remaining trees was calculated. The sequence alignment was deposited at TreeBase (submission ID: 30414). Branches that received bootstrap supports for ML and Bayesian posterior probabilities (BPP) greater than or equal to 75% (ML) and 0.95 (BPP) were considered significantly supported.

## 3 Results

### 3.1 Phylogeny

The ITS dataset comprised 229 fungal collections representing approximately 81 taxa of the genus *Cortinarius*. ModelFinder suggested that GTR + I + G was the best-fit model of nucleotide evolution for BI. The Bayesian analysis resulted in a concordant topology with an average standard deviation of split frequencies of 0.005575. The ML and BI analyses resulted in nearly identical topologies, and thus only the ML tree is presented with the bootstrap supports for ML and BPP when they were greater than or equal to 50% and 0.90, respectively.

**Figure 1:**
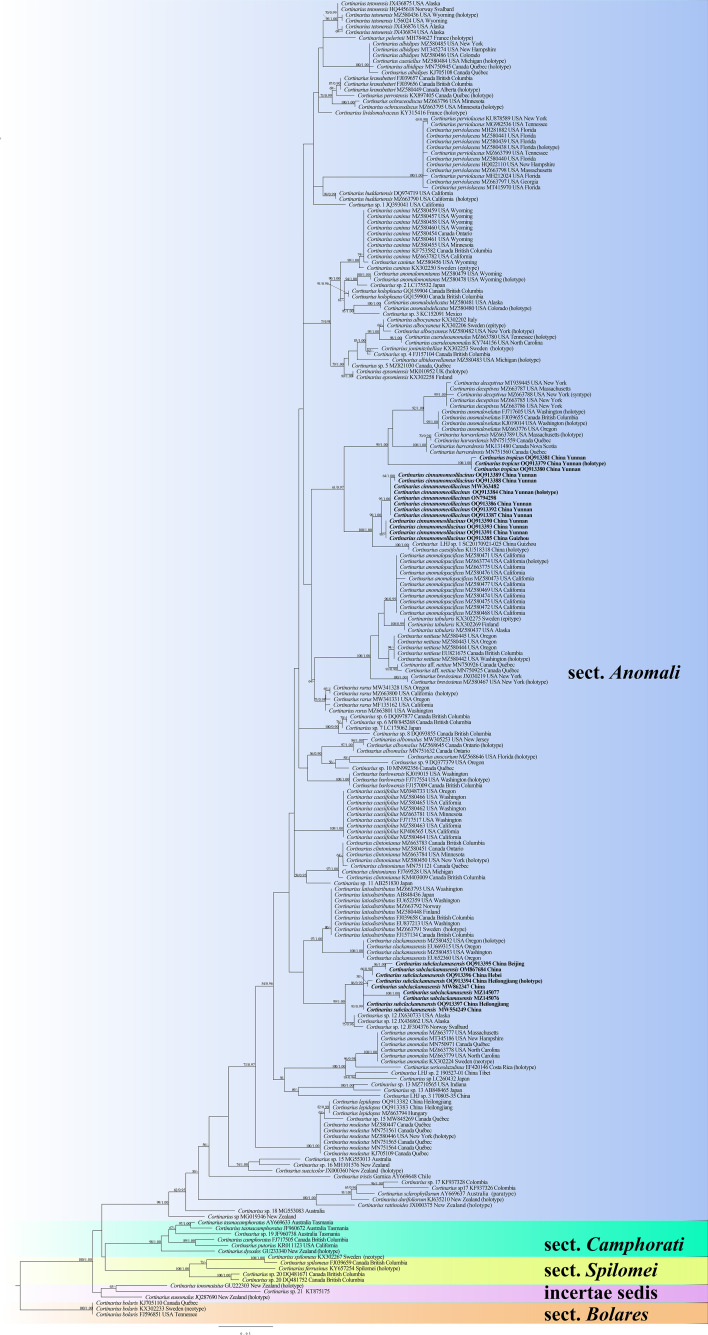
Maximum likelihood (ML) tree illustrating the phylogeny of *Cortinarius* section *Anomali* based on ITS sequences. Branches are labeled with ML bootstrap > 50% and Bayesian posterior probabilities (BPP) > 0.90, respectively. New species are highlighted in bold.

Our phylogeny, which is inferred from ITS sequences (**Figure 1**), is similar to those of Dima et al (Dima et al., 2016; Dima et al., 2021). The phylogenetic analysis showed five sections, and each section formed separate monophyletic lineages with strong statistical support. Section *Anomali* formed a distinct highly supported clade (ML = 99 and BPP = 1) and was separated from other sections. Three new species, namely, *C. cinnamomeolilacinus* (ML = 98 and BPP = 1), *C. subclackamasensis* (ML = 86 and BPP = 0.99), and *C. tropicus* (ML = 100 and BPP = 1), nested within the sect. *Anomali* clade, and formed an independent lineage with high statistical supports, respectively. It is worth noting that collections of *C. cinnamomeolilacinus* had genetic distances in our phylogeny. However, there were only four base pair differences between them, which were below 1.5% nucleotide differences in the ITS regions. Morphologically, there were no obvious differences of these *C. cinnamomeolilacinus* collections.

### 3.2 Taxonomy

**Figure 2:**
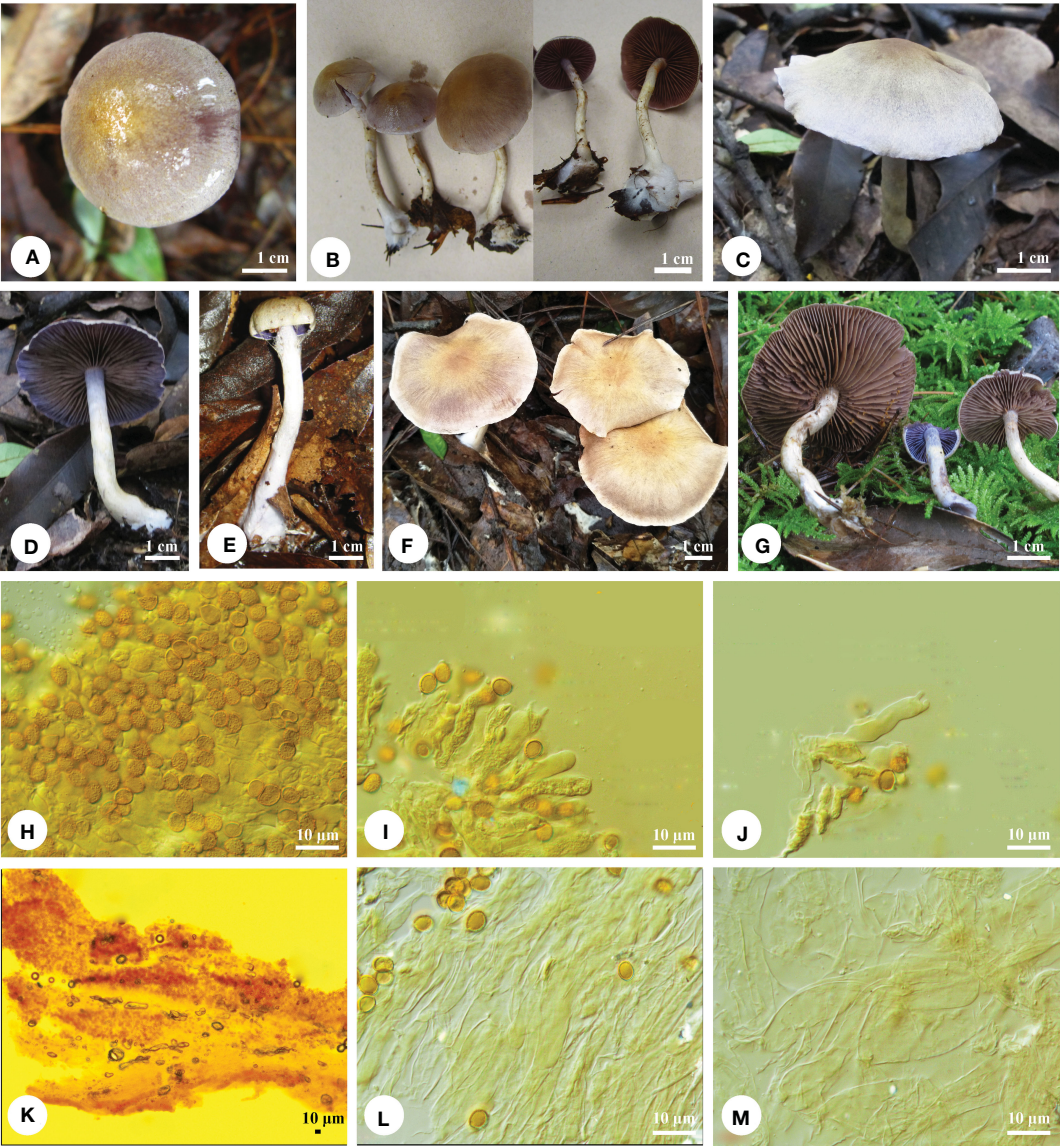
Basidiomata and microscopic structures of *Cortinarius cinnamomeolilacinus*. **(A-G)** Basidiomata **(A, B)** TCWH007; **(C, D)** Li 180825-21; **(E)** LLLJ20170805-002; **(F)** tcmb005; **(G)** 180825-21), **(H)** Basidiospores, **(I-J)** Basidia and basidioles, **(K)** Pileipellis, **(L)** Hypodermium of pileipellis, and **(M)** Context hyphae.

***Cortinarius cinnamomeolilacinus*** Q.Y. Zhang, Jing Si & Hai J. Li, sp. nov. **Figure 2**.

MycoBank: 848613

*Diagnosis*. — This species is characterized by its small basidiomata, more or less hygrophanous, lilac pileal surface with cinnamon buff to brownish cinnamon center, and pale gray to whitish toward margin, subglobose to broadly ellipsoid basidiospores (7−8.5 × 5.8−6.8 μm); it is gregarious on ground dominant with Fagaceae or Pinaceae.

*Type*. — China, Yunnan Province, Baoshan, Tengchong, Wuhe Town, Lushan, Alt: 1989 m, N: 24°54′06.98″, E: 98°36′30.37″, on ground dominant with Fagaceae, 8 August 2016, TCWH 007 (holotype), and GenBank accession number for ITS: OQ913384.

*Etymology*. — *cinnamomeolilacinus* refers to its more or less lilac pileal surface with a cinnamon center.

*Habitat and distribution*. — Scattered or gregarious on ground dominant with Fagaceae or Pinaceae. Currently, it is only found in tropical Guizhou and Yunnan (nine collections) in summer and autumn.

*Macrostructures*. — Basidiomata small. Pileus 15−52 mm in diam., hemispheric when young, then convex to plano-convex, some with a low umbo, margin narrowly when young, surface smooth to finely felty, color evenly pale silvery gray to lilac [15A(2−4)], the disc slightly more brownish, cinnamon buff, cinnamon to brownish cinnamon [5B(4−5)], pale gray to whitish (1A1−1B1) toward margin, even to rugulose, hygrophanous. Lamellae adnate with decurrent tooth to slightly adnexed, close, violet (16A4−16A5) when young, then grayish violet [16B4−16C5], pale silvery gray to pale drab gray (16B2−16D2), finally pale brown to cinnamon [6D(4−8)]. Stipe 30- to 70- mm long, apex 4−6 mm in diam., and base 5−10 mm in diam., usually clavate to cylindrical, even or slightly bulbous at base, fragile, shiny, covered with white fibrillose, apex violet [16A(4−5)] when young, later pale brownish (6B2−6D2), other part pale silvery gray [15A(2−3)], pale white veil (1A2) usually sparse, forming yellow [3A(3−4)] floccose-girdles on the stipe, often becoming pale yellow [4B(3−5)], sometimes indistinct, basal mycelium white (1A1). Context in pileus solid, firm, sometimes hollow in stipe, pale silvery gray [16B4−16C5] when fresh, finally pale brown to cinnamon in age [6D(4−8)]. Odor and taste of context strongly fungoid.

*Microstructures*. — Basidiospores [150/5/5] (6.8−) 7−8.5 (−8.8) × (5.5−) 5.8−6.8 (−7) μm, av. 7.7 × 6.1 μm, *Q* = 1.22−1.29, av. *Q* = 1.26, subglobose to broadly ellipsoid, buff to cinnamon-buff, coarsely verrucose, non-dextrinoid. Basidia 4-spored, 29−33 × 6−7 μm, clavate, colorless, or yellowish. Lamella trama hyphae smooth, parallel, 5- to 12- μm wide. Lamellae edge fertile, with some small clavate sterile cells. Pileipellis duplex: Epicutis thin to ± well developed, hyphae ± cylindrical, moderately to strongly interwoven, 5- to 8- μm wide, hyaline or yellowish, smooth to encrusted; hypocutis ± cellular or hyphae more interwoven and radially arranged, cylindrical to enlarged, hyaline to yellowish, smooth to encrusted, (3.5) 4−18 (20) μm wide. Clamp connections present.

*Remarks*. — *Cortinarius albomalus* Liimat. & Niskanen and *C. cinnamomeolilacinus* have similar basidiomata. However, *C. albomalus* has smaller basidiospores (6.5−7.5 × 5.5−6 µm) and is distributed in North America (Dima et al., 2021; Liimatainen and Niskanen, 2021). *Cortinarius pastoralis* Soop, H. Lindstr., Dima, Niskanen, Liimat. & Kytöv. and *C. cinnamomeolilacinus* share pale buff or brownish pilei, but *C. pastoralis* has larger basidiospores (8.5−9 × 7−8 μm; Dima et al., 2016). *Cortinarius albocyaneus* Fr. is similar to *C. cinnamomeolilacinus* with grayish ochraceous to whitish pilei, but *C. albocyaneus* has larger basidiomata (40−70 mm) and basidiospores (8.5−9.0 × 6.0−7.5 μm), is found in Europe, and is common in northeastern North America (Dima et al., 2021). *Cortinarius anomalovelatus* Ammirati, Berbee, E. Harrower, Liimat. & Niskanen has grayish-to-white basidiomata and subglobose to broadly ellipsoid basidiospores, which are similar to those of *C. cinnamomeolilacinus*, but *C. anomalovelatus* has a heavier universal veil and a grayish-blue to violet pileal surface, and is usually found in western North America (Dima et al., 2021).

*Additional specimens (paratypes) examined*. — China, Guizhou Province, Liupanshui, Lingshan Temple, on ground of Fagaceae, Alt: 1929 m, N: 26°37′35.94″, E: 104°48′54.95″, October 15, 2016, Li 161015-10; Yunnan Province, Baoshan, Longling County, Longjiang Town, Laohuangtian, on ground dominant with Fagaceae, Alt: 1773 m, N: 24°41′31.01″, E: 98°42′55.24″, 5 August 2017, LLLJ 20170805-002; Longyang District, Wafang Town, Dapingdi, on ground of *Pinus yunnanensis*, Alt: 1921.9 m, N: 25°21′35.82″, E: 99°4′45.73″, 6 August 2018, LYWF015; Tengchong, Mangbang Town, Hongdoushu, on ground dominant with Fagaceae, Alt: 1772 m, N: 24°54′52″, E: 98°37′59″, 8 August 2018, tcmb 005; Menglian Town, Xiamenglian Village, on ground of mixed forests composed of Fagaceae and *P. yunnanensis*, 5 August 2014, Li 140805-18; Chuxiong Yi Autonomous Prefecture, Zixi Mountain, near King Baotou, on ground of mixed forest composed of Fagaceae and *P. yunnanensis*, Alt: 1926 m, N: 25°1′3″, E: 101°24′7″, 25 August 2018, Li 180825-21; Yuxi, Huaning County, on ground dominant with Fagaceae, 8 September 2013, Li 130908-29; Zhaotong, Weixin County, Miaogou Town, Zhashigou Village, on ground dominant with Fagaceae, Alt: 1450 m, N: 27°47′6″, E: 104°49′18″, 22 September 2017, WX 20170922065.

**Figure 3:**
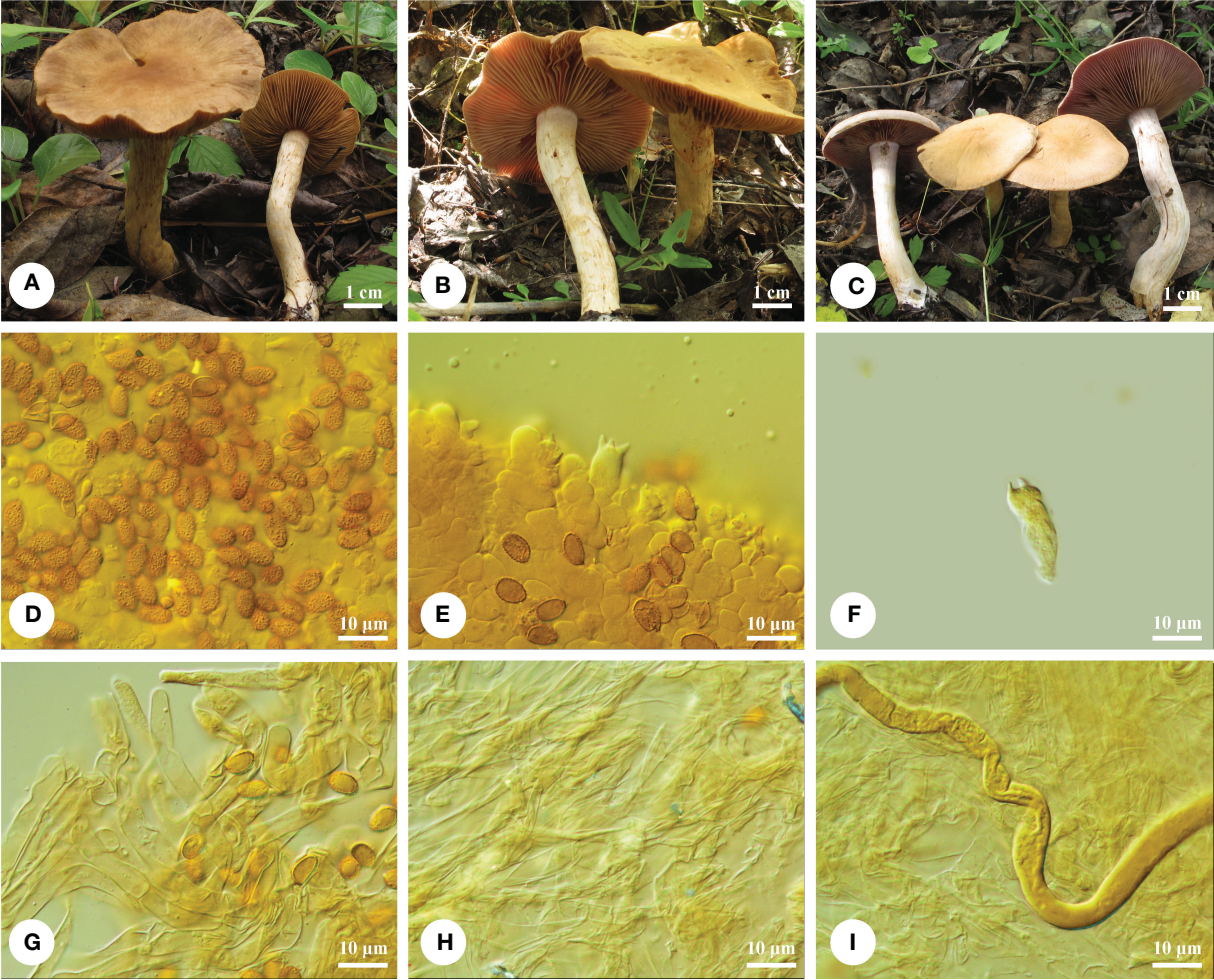
Basidiomata and microscopic structures of *Cortinarius subclackamasensis*. **(A-C)** Basidiomata (**A** Li 170818-16; **B** Li 170818-02; **C** Li 170818-01), **(D)** Basidiospores, **(E)** Hymenium in trama, **(F)** One basidium, **(G)** Epicutis of pileipellis, **(H)** Hypodermium of pileipellis, and **(I)** Context hyphae.

***Cortinarius subclackamasensis*** Q.Y. Zhang, Jing Si & Hai J. Li, sp. nov. **Figure 3**.

MycoBank: 848614

*Diagnosis*. — This species notably has small- to medium-sized basidiomata, buff-yellow and plano-convex pilei, ellipsoid to oblong ellipsoid basidiospores (9.5−10.8 × 5.8−6.8 μm); it is gregarious on ground of *Salix* or other deciduous trees and distributed in temperate China.

*Type*. — China, Heilongjiang Province, Huzhong, Huzhong Forest Farm, near Dongfanghong Line 31, on ground of *Salix*, 18 August 2017, Li 170818-16 (holotype), GenBank accession number for ITS: OQ913394.

*Etymology*. — *Subclackamasensis* refers to its morphological similarity to *C. clackamasensis.*

*Habitat and distribution*. — Scattered or gregarious on ground of *Salix* or other deciduous trees. Currently, it is found in Northeast and North China in summer.

*Macrostructures*. — Basidiomata small to medium sized. Pileus 30−60 mm in diam., hemispheric to subhemispheric when young, becoming plano-convex, then depressed at center and wrinkled, margin sharp sometimes waves; surface moist to hygrophanous when young, with fibrous veil remnants; buff yellow (4A4), to light honey yellow (4/5B4), somewhat lighter olivaceous buff (4C4) at margin, finely radially striate with age. Lamellae adnexed-emarginate, moderately crowded to crowded, vinaceous (9F6) or peach (6A6) when young, somewhat darkening on manipulation, edge even, somewhat lighter. Stipe 40- to 60- mm long, 5- to 10- mm thick above, qual to somewhat clavate, white, and cream with age. Universal veil usually sparse, thin, and somewhat glossy, cream (4A2/3) to light yellow (4A4), flocculose or forming a week girdle on the stipe. Context rather thick, especially in pileus center, brittle, weakly light honey yellow (4/5B4) with age. Odor and taste of context strongly fungoid.

*Microstructures*. — Basidiospores [90/3/3] 9.5−10.8 (−11) × (5.5−) 5.8−6.8 μm, av. 9.9 × 6.3 μm, *Q* = 1.56−1.60, av. *Q* = 1.59, ellipsoid to oblong ellipsoid, weekly to moderately, but distinctly verrucose, indextrinoid to weakly dextrinoid. Basidia 4-spored, 22−25 × 8−11 μm, clavate, colorless or yellowish. Lamella trama hyphae smooth, parallel, 4- to 12- μm wide. Lamellae edge fertile, with some small clavate sterile cells. Pileipellis duplex: Epicutis about 27- to 32 -μm thick, hyphae (6−) 11- to 16- μm wide, in upper part loosely entangled; hypocutis distinct well-developed, colorless or yellowish, irregular, interwoven, with some intracellular pigments, 18−28.5 μm in diam. Clamp connections present.

*Remarks*. — Phylogenetically, *C. latiodistributus* Dima, Ammirati, Niskanen, Kytöv., Liimat. & Brandrud, *C. clackamasensis* Ammirati, Liimat., Niskanen & Dima, and *Cortinarius* sp. 12 are closely related to *C. subclackamasensis*. *Cortinarius latiodistributus* has violet to pallid brown pilei, and shorter basidiospores (7−9.5 μm). *Cortinarius clackamasensis* has wider basidiospores (9−11 × 6.5−7.5 μm, av. 9.7 × 6.5 μm, *Q* = 1.4−1.5), grows on the ground under mixed conifers composed of *Picea*, *Pinus*, and *Abies*, and is distributed in the US (Dima et al., 2021). Similar to *C. subclackamasensis*, *C. clintonianus* Peck, and *C. anomalopacificus* Bojantchev, Liimat., Niskanen, Dima & Ammirati have yellowish and plano-convex basidiomata. However, the basidiospores in *C. clintonianus* and *C. anomalopacificus* are shorter (6.7−8.1 × 5.6−6.3 μm *vs.* 6.5−7 × 5−6 μm; Dima et al., 2021).

*Additional specimens (paratypes) examined*. — China, Beijing, Mentougou District, Xiaolongmen National Forest Park, 30 August 2017, BJMTG20170830-34; Hebei Province, Baoding, Fuping County, Dongxiaguan Town, Zhujiaying Village, Tianshengqiao, 22 August 2019, 20190822-11; Heilongjiang Province, Huzhong, Huzhong forest farm, near Dongfanghong Line 31, on ground of *Salix*, 18 August 2017, Li 170818-01 and Li 170818-02.

**Figure 4:**
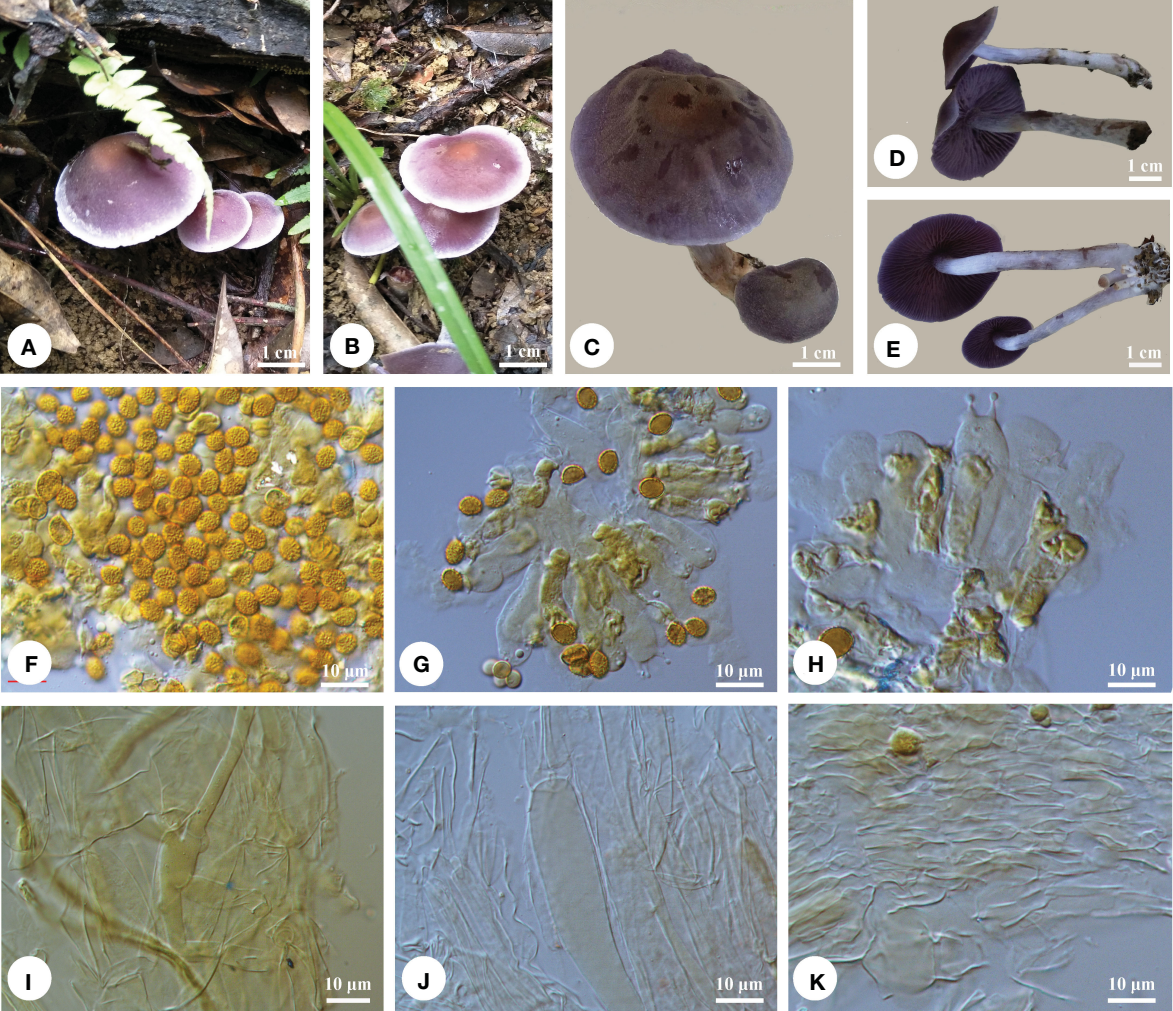
Basidiomata and microscopic structures of *Cortinarius tropicus*. **(A-E)** Basidiomata (tcqushi 006, Holotype), **(F)** Basidiospores, **(G-H)** Basidia and basidioles, **(I)** Epicutis of pileipellis, **(J)** Hypodermium of pileipellis, and **(K)** Context hyphae.

***Cortinarius tropicus*** Q.Y. Zhang, Jing Si & Hai J. Li, sp. nov. **Figure 4**.

MycoBank: 848616

*Diagnosis*. — This species notably has small basidiomata, violet to dark violet pileal surface with fibrillose disc and nearly glabrous, cream to pale violet margin, subglobose to broadly ellipsoid basidiospores (6−8 × 5−6 μm); it is scattered or gregarious on ground dominant with Fagaceae.

*Type*. — China, Yunnan Province, Baoshan, Tengchong, Qushi Town, Qingqiao Village, Alt: 1490 m, N: 25°17′17″, E: 98°35′26″, on ground dominant with Fagaceae, 6 August 2016, tcqushi 006 (holotype), GenBank accession number for ITS: OQ913379.

*Etymology*. — *tropicus* refers to its tropical distribution in Southwest China.

*Habitat and distribution*. — Scattered or gregarious on ground dominant with Fagaceae. Currently, it is only found in tropical Yunnan (three collections) in summer.

*Macrostructures*. — Basidiomata small. Pileus 20−45 mm in diam., hemispheric to subhemispheric when young, becoming obtusely conical, conico-convex, broadly umbonate, pale brown [5D(4−5)] at center and gradually paler toward margin when young, violet (17B8) to dark violet (17F8) when mature, disc grayish violet (15E5) to dark violet (17F8), margin cream (4A2/3) to pale violet [16A(2−3)], fibrillose at disc and nearly glabrous near margin, somewhat hygrophanous, margin decurved. Lamellae adnexed-emarginate, subclose, purplish to pale violet lilac [16D(4−6)], finally ± pale brown to cinnamon (16D3-16E3). Stipe 50- to 80- mm long, apex 3 to 5 mm in diam., base 5−10 mm in diam., enlarged, narrowly clavate, surface grayish (16B2) with dark violet [16F(3−4)] streaks or silvery violet (16A3) on upper half and watery brownish below, veil usually sparse, forming floccose-girdles on the stipe, often at first brownish (6B2-6C2) then becoming pale yellow [4A(2-3)], sometimes indistinct, and basal mycelium whitish. Context in pileus solid, firm, violet to silvery violet [16C(4−6)] when fresh, finally pale brown to cinnamon (16D4−16F4) in age. Odor and taste of context strongly fungoid.

*Microstructures*. — Basidiospores [90/3/3] 6−8 × 5−6 μm, av. 7.4 × 5.9 μm, *Q* = 1.21−1.29, av. *Q* = 1.25, subglobose to broadly ellipsoid, buff to cinnamon-buff, coarsely verrucose, non-dextrinoid to slightly dextrinoid. Basidia 4-spored, 30−42 × 7−11 μm, clavate, colorless or yellowish. Lamella trama hyphae hyaline, smooth, parallel, 5- to 13- μm wide. Pileipellis duplex: Epicutis well developed, hyaline or yellow to ochraceous, smooth, cylindrical, radially arranged, ± interwoven, 4−10 μm wide; hypocutis distinct, colorless or yellowish, smooth to slightly encrusted, cylindrical to enlarged, radially oriented, ± interwoven, (4)6- to 15(17)- μm wide. Clamp connections present.

*Remarks*. — *Cortinarius perviolaceus* Murrill is easily confused with *C. tropicus* because of its violet pilei, but *C. perviolaceus* has smaller basidiomata (8−22 mm *vs.* 20−45 mm) and wider basidiospores (6−6.5 μm *vs.* 5−6 μm; Dima et al., 2021). *Cortinarius anomalus* has similar pilei with blue tinge when young and larger basidiospores (8−9 × 6−7 μm; Dima et al., 2016). *Cortinarius anomalovelatus*, *C. deceptivus* Kauffman, and *C. harvardensis* L. Nagy, Dima & Ammirati formed a sister group with *C. tropicus*. Compared with the new species, *C. anomalovelatus* has wider basidiospores (6.3−7 μm *vs.* 5−6 μm), *C. deceptivus* produces larger basidiospores (7.8−9.3 × 6−7.4 μm), and *C. harvardensis* has bluish to violet pileus, lamellae, and stipe, and slightly bigger basidiospores (7.5−8.5 × 5.5−6.5 μm; Dima et al., 2021).

*Additional specimens (paratypes) examined*. — CHINA. Yunnan Province, Dehong Dai and Jingpo Autonomous Prefecture, Mang City, Jiangdong Town, Daxinzhai, Alt: 1619 m, N: 24°27′54″, E: 98°18′56″, on ground dominant with Fagaceae, 28 July 2015, Li 150728-56 and Li 150728-63.

## 4 Discussion

*Cortinarius*, the largest agaric genus worldwide, contains important ectomycorrhizal fungi, among which sect. *Anomali* represents a monophyletic, species-rich group of this genus (Dima et al., 2016; Garnica et al., 2016; Soop et al., 2019; Dima et al., 2021; Liimatainen and Niskanen, 2021; Xie, 2022). Species recognition based on morphology is difficult in *Cortinarius* lineages due to overlapping characteristics and variations within species. Notably, the basidiospore size helps identify some species when used in correlation with other characteristics (Frøslev et al., 2006; Niskanen et al., 2013; Liimatainen et al., 2014; Liimatainen et al., 2015; Niskanen et al., 2016).

According to our phylogenetic analysis, sect. *Anomali* indicated a widely distributed lineage of *Cortinarius* in both the northern and southern Hemispheres. Furthermore, certain patterns of distribution correlated with ecology and plant hosts. *Cortinarius albocyaneus* exhibited regional patterns of distribution with conifers in northern Michigan and northern Europe. Several species, including *Cortinarius brevissimus* Peck, *C. caeruleoanomalus* Dima, Matheny, K. Hughes & Ammirati, *C. deceptivus*, *C. harvardensis*, *C. modestus* Rob. Henry, *C. perrotensis* A. Paul, Matheny & Lebeuf, and *C. perviolaceus*, occurred in hardwood, mixed hardwood conifer, and/or conifer forests in eastern North America. *Cortinarius rarus* Bojantchev, Ammirati, Parker, Liimat., Niskanen & Dima displayed regional patterns of distribution associated with mountain conifer forests (Dima et al., 2016; Dima et al., 2021).

Current studies related to this genus have indicated significant regional variations. Classical European species were examined and typified by Dima et al. (2016) before species from other parts of the world were studied. In sect. *Anomali*, more than 50 binomials were introduced in the last century, mainly from Europe, with fewer from elsewhere, but only about 20% of these names have been in general use (Dima et al., 2021). China is geographically located in East Asia and has a land area comparable with that of the entire Europe, various climate types ranging from the temperate to the tropical climate, as well as a complex and diverse habitat, which provides an ideal place for the survival of *Cortinarius* species. However, the resource investigation and taxonomic research on *Cortinarius* have not yet been extensively carried out in China. Currently, there have been reports of a total of 163 taxa of the genus in China, with only three species in the sect. *Anomali*, viz. *C. caninus* (Fr.) Fr., *C. albocyaneus*, and *C. tabularis* (Fr.) Fr. Therefore, the collection of more samples from China and exploration of multigene phylogeny are urgently needed to systematically elucidate the diversity of *Cortinarius* s.l.

## Data availability statement

The datasets presented in this study can be found in online repositories. The names of the repository/repositories and accession number(s) can be found below: https://www.ncbi.nlm.nih.gov/nuccore/OQ913379,OQ913380,OQ913381,OQ913382,OQ913383,OQ913384,OQ913385,OQ913386,OQ913387,OQ913388,OQ913389,OQ913390,OQ913391,OQ913392,OQ913393,OQ913394,OQ913395,OQ913396,OQ913397,OQ920003,OQ920004,OQ920005.

## Author contributions

Q-YZ, CJ, H-MZ, Z-YM, Y-ZZ, J-QL, JS, and H-JL designed the research and contributed to data analysis and interpretation. Q-YZ, CJ, H-MZ and Z-YM prepared the samples and drafted the manuscript. Y-ZZ, J-QL, Z-YM, JS, and H-JL conducted the molecular experiments and analyzed the data. JS and H-JL discussed the results and edited the manuscript. All authors contributed to the article and approved the submitted version.
